# Response to First-Line Chemotherapy in Patients with Non-Small Cell Lung Cancer According to RRM1 Expression

**DOI:** 10.1371/journal.pone.0092320

**Published:** 2014-03-19

**Authors:** Xiaopeng Dong, Yingtao Hao, Yucheng Wei, Qiuwei Yin, Jiajun Du, Xiaogang Zhao

**Affiliations:** 1 Department of Thoracic Surgery, Second Hospital of Shandong University, Jinan, China; 2 Department of Thoracic Surgery, Affiliated Hospital of Qingdao University Medical College, Qingdao, China; 3 Department of Thoracic Surgery, Qilu Hospital of Shandong University, Jinan, China; 4 Department of Thoracic Surgery, Shandong Provincial Hospital, Jinan, China; University of Algarve, Portugal

## Abstract

**Background:**

The response to cytotoxic chemotherapy varies greatly in patients with advanced non-small cell lung cancer (NSCLC), and molecular markers may be useful in determining a preferable therapeutic approach for individual patients. This retrospective study was performed to evaluate the predictive value of ribonucleotide reductase regulatory subunit M1 (RRM1) on the therapeutic efficacy of platinum-based chemotherapy in patients with NSCLC.

**Methods:**

Patients with advanced NSCLC who received platinum doublet chemotherapy (n = 229) were included in this retrospective study, and their clinical outcomes were analyzed according to RRM1 expression.

**Results:**

In patients receiving gemcitabine-based therapy, the disease control rate (DCR) and progression-free survival (PFS) of patients with RRM1-negative tumors were significantly higher than in patients with RRMI-positive tumors (*P = *0.041 and *P = *0.01, respectively), and multivariate analysis showed that RRM1 expression was an independent prognostic factor (*P = *0.013). No similar differences were found in patients receiving docetaxel- or vinorelbine-based therapy. In RRM1-positive patients, the DCRs for docetaxel and vinorelbine were higher than for gemcitabine (*P = *0.047 and *P = *0.047, respectively), and docetaxel and vinorelbine showed a longer PFS than gemcitabine-based chemotherapy (*P = *0.012 and *P = *0.007). No similar differences were found among patients with RRM1-negative tumors.

**Conclusions:**

Negative RRM1 expression in advanced NSCLC is associated with a higher response rate to gemcitabine-based chemotherapy. In patients with RRM1-positive tumors, docetaxel and vinorelbine showed a higher therapeutic efficacy than gemcitabine-based therapy. Additional prospective studies are needed to investigate the predictive meaning of RRM1 in the response to chemotherapy.

## Introduction

Platinum-based chemotherapy is considered the main therapeutic approach for advanced non-small cell lung cancer (NSCLC) [1], 2. However, the selection of chemotherapeutic agents is primarily based on the clinician’s experience and preference, and studies have shown a great deal of variability with respect to their therapeutic efficacy and toxicity. Even with newly developed chemotherapy regimens, the prognosis of patients with advanced NSCLC remains dismal [3]–[5].

At present, promising results on the utility of molecular markers in predicting efficacy of cytotoxic therapy in NSCLC have been reported. Excision repair cross-complementation group 1 (ERCC1) was shown to be associated with the response to platinum-based chemotherapy [6]–[8], and in another recent study, taxane-based therapies showed a higher disease control rate (DCR) and longer progression-free survival (PFS) than gemcitabine in patients with epidermal growth factor receptor (EGFR) mutations [9]. These studies suggest that the tumor biology and the response to cytotoxic chemotherapy vary greatly among NSCLC patients, and individualized therapies may help reduce the resistance to chemotherapeutic agents.

Ribonucleotide reductase regulatory subunit M1 (RRM1) is a molecule involved in DNA synthesis and damage repair. Preclinical studies have shown that RRM1 is involved in sensitivity to gemcitabine in NSCLC [10], [11]. Lower RRM1 expression was associated with a high response rate to platinum agents and gemcitabine, and patients with high expression of RRM1 showed a decreased response to gemcitabine therapy [12]–[15]. However, in other reports, RRM1 was either not associated or was inversely associated with the survival of NSCLC patients receiving gemcitabine-containing regimens [16], [17]. Therefore, the correlation between RRM1 expression and the response to chemotherapy is still uncertain.

In the present study, we reviewed 229 patients with advanced NSCLC who had received platinum-based doublet chemotherapy as a first-line therapy, and evaluated their clinical outcomes according to RRM1 expression.

## Patients and Methods

### Ethics Statement

This retrospective study was approved by the ethics committee of second hospital of Shandong university. And all patient records were anonymized and de-identified prior to analysis.

### Patients

In this retrospective analysis, 680 patients diagnosed with advanced NSCLC between 2007 and 2010 were screened, 325 of whom had received carboplatin-based doublet chemotherapy as a first-line treatment. A cohort of 229 patients, for whom clinical records and computed tomography (CT) scans were complete and tumor specimens were available to screen for RRM1 expression, was selected. Histological type was determined according to the World Health Organization criteria. During the treatment period, a chest CT scan was taken every 6–8 weeks, and independent reviews of these CT scans were performed in this retrospective study to confirm the response to therapy and to assess disease progression. The treatment response was classified as progressive disease (PD), stable disease (SD), partial response (PR), or complete response (CR), according to RECIST (Response Evaluation Criteria in Solid Tumors). Patients showing a CR or PR were regarded as responders. The DCR included patients with CR, PR, and SD lasting longer than three months. PFS was the time between the first day of treatment and the first sign of disease progression or death.

### RRM1 Expression Analysis

Immunohistochemistry was performed using 5 μm-thick sections from paraffin-embedded tissue blocks and a Bond Polymer Intense Detection System (VisionBioSystems, Vic, Australia), according to the manufacturer’s instructions. As a negative control, the same immunohistochemical staining protocol was used except the specific primary antibody (ProteinTech Group, Chicago, USA) was replaced with distilled water. Formalin-fixed, paraffin-embedded human colonic adenocarcinoma tissue was used as a positive control.

Five fields at 400× magnification were selected for each section to assess immunoreactivity. RRM1 immunoreactivity was evaluated semi-quantitatively based on the staining intensity and the proportion of positively staining cells by two independent observers blinded to patient status. The proportion of staining was scored from 0 to 3 as follows: diffuse, ≥50% positive (score 3); regional, 10–49% positive (score 2); focal, 1–9% positive (score 1); and negative, <1% positive (score 0). The intensity of staining was also scored from 0 to 3 (0, absent; 1, weak; 2, moderate; 3, intense). The immunoreactive score for each sample was determined by multiplying the two individual scores. A score of ≥9 was defined as a positive/high expression, and a score of <9 was considered a negative/low expression.

### Statistical Analysis

Statistical analyses of categorical variables, including response rate (RR) and DCR, were performed using Fisher’s exact test. Comparisons of the mean between different groups were calculated using the Student’s t-test. The median duration of PFS was calculated using the Kaplan-Meier method. Multivariate analyses were performed using Cox regression analysis for PFS to identify independent factors. Two-sided *P*-values of less than 0.05 were considered significant. All analyses were performed using SPSS 17.0 for Windows.

## Results

### Patient Characteristics and RRM1 Expression

A total of 229 NSCLC patients were included in the study. The ages ranged from 39 to 75 years (median age, 61 years), and 127 patients (55.5%) were male. The majority of the tumors were adenocarcinoma (112 patients, 48.9%) and 123 patients had stage IV disease (53.7%). All patients received carboplatin-based doublet chemotherapy as a first-line treatment. Gemcitabine, docetaxel, and vinorelbine regimens were administered in 81 (35.4%), 77 (33.6%) and 71 (31.0%) cases, respectively, and the choice of regimen was made by the responsible clinician (Table 1). Of the 229 tumors, 146 (63.8%) were negative for RRM1 expression, and 83 (36.2%) were positive for RRM1 (Table 1).

**Table 1:** Basic characteristics of NSCLC patients.

Characteristics	No	%
No. of patients	229	
Age (median years)	61	Range, 39–75
Gender		
Male	127	55.5
Female	102	44.5
History of smoking		
Never smoker	130	56.8
Smoker	99	43.2
Histology		
Adenocarcinoma	112	48.9
Squamous cell carcinoma	67	29.3
Others	50	21.8
Stage		
IIIB	106	46.3
IV	123	53.7
RRM1		
Negative	146	63.8
Positive	83	36.2
Chemotherapeutic regimen		
Gemcitabine and carboplatin	81	35.4
Docetaxel and carboplatin	77	33.6
Vinorelbine and carboplatin	71	31.0

The relationship between patient characteristics and chemotherapy regimens according to RRM1 expression was analyzed. The patient characteristics were similar among patients receiving gemcitabine-, docetaxel-, and vinorelbine-based therapies (Table 2).

**Table 2:** Characteristics of patients receiving chemotherapeutic regimens according to RRM1 expression.

Characteristics	RRM1-negative			*P-value*	RRM1-positive			*P-value*
	Gemcitabine	Docetaxel	Vinorelbine		Gemcitabine	Docetaxel	Vinorelbine	
No. of patients	52	50	44		29	27	27	
Age (years)	58	62	61	>0.05#	60	58	63	>0.05#
Gender								
Male	29 (55.8%)	28 (56.0%)	25 (56.8%)	>0.05*	16 (55.2%)	15 (55.6%)	14 (51.2%)	>0.05*
Female	23 (44.2%)	22 (44.0%)	19 (43.2%)		13 (44.8%)	12 (44.4%)	13 (48.8%)	
Smoking history								
Never smoker	30 (57.7%)	31 (62.0%)	25 (56.8%)	>0.05*	15 (51.7%)	15 (55.6%)	14 (51.9%)	>0.05*
Smoker	22 (42.3%)	19 (38.0%)	19 (43.2%)	>0.05*	14 (48.3%)	12 (44.4%)	13 (48.1%)	>0.05*
Histology								
Adenocarcinoma	24 (46.2%)	25 (50.0%)	22 (50.0%)	>0.05*	14 (48.3%)	14 (51.9%)	13 (48.2%)	>0.05*
Squamous cell carcinoma	17 (32.7%)	15 (30.0%)	12 (27.3%)		8 (27.6%)	8 (29.6%)	7 (25.9%)	
others	11 (21.1%)	10 (20.0%)	10 (22.7%)		7 (24.1%)	5 (18.5%)	7 (25.9%)	
Stage								
IIIB	24 (46.2%)	22 (44.0%)	20 (45.5%)	>0.05*	14 (48.3%)	12 (44.4%)	14 (51.9%)	>0.05*
IV	28 (53.8%)	28 (56.0%)	24 (54.5%)		15 (51.7%)	15 (55.6%)	13 (48.1%)	

*Based on Fisher’s exact test.

# Based on Student’s *t*-test.

### Tumor Response and PFS According to RRM1 Expression

In the 229 patients, 3 CRs, 77 PRs, 101 SDs, and 48 PDs were observed, for an overall RR and DCR of 34.9% and 79.0%, respectively. There were no differences in the RR and DCR between patients with RRM1-negative tumors and those with RRM1-positive tumors. However, in patients receiving gemcitabine-based therapy, the DCR of RRM1-negative patients was significantly higher than that of RRM1-positive cases (78.8% vs. 55.2%, *P = *0.041). No similar difference was found in patients receiving docetaxel- or vinorelbine-based therapy (Table 3).

**Table 3:** Response to chemotherapy according to RRM1 expression.

	Gemcitabine and carboplatin						Docetaxel and carboplatin						Vinorelbine and carboplatin					
	CR	PR	SD	PD	RR	DCR	CR	PR	SD	PD	RR	DCR	CR	PR	SD	PD	RR	DCR
RRM1																		
Negative	1	18	22	11	19	41*	1	17	26	6	18	44	1	14	21	8	15	36
Positive	0	7	9	13	7	16* #	0	10	12	5	10	22#	0	11	11	5	11	22#

*Based on Fisher’s exact test. In patients receiving gemcitabine-based therapy, the DCR of RRM1-negative patients was higher than RRM1-positive patients (*P = *0.041).

# Based on Fisher’s exact test. In patients with RRM1-positive tumors, the DCRs for docetaxel and vinorelbine were higher than for gemcitabine-based therapy (*P = *0.047 and *P = *0.047, respectively).

**Figure 1:**
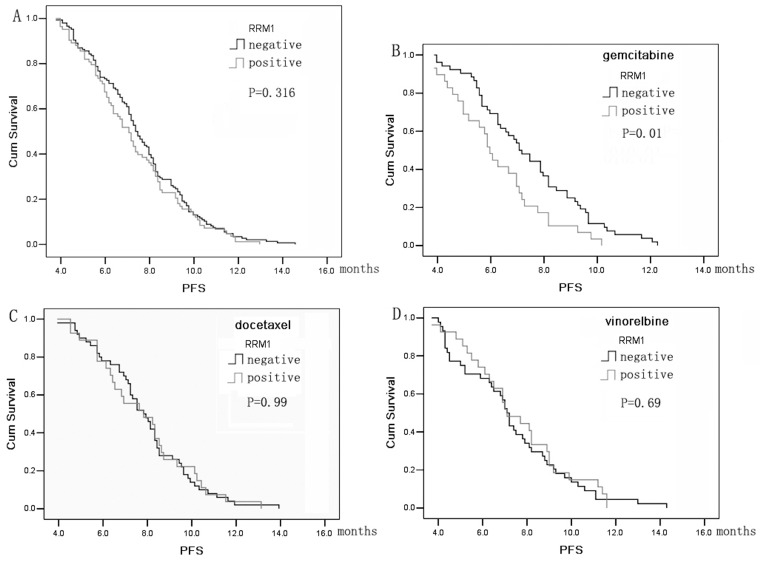
Kaplan-Meier curve of progression-free survival (PFS) according to ribonucleotide reductase M1 (RRM1) expression. (A) PFS for all patients with negative or positive RRM1 expression. (B) PFS for patients receiving gemcitabine-based therapy. (C) PFS for patients receiving docetaxel-based therapy. (D) PFS for patients receiving vinorelbine-based therapy.

The median PFS was 8.7 months (95% confidence interval (CI): 8.5–9.0 months) in all patients. No difference in PFS was found between patients with RRM1-negative tumors and those with RRM1-positive tumors (8.9 months vs. 8.5 months, *P = *0.316) (Fig. 1A). However, in patients receiving gemcitabine-based therapy, the PFS of RRM1-negative patients was significantly higher than that of RRM1-positive patients (8.8 months vs. 7.6 months, *P = *0.01) (Fig. 1B). No similar difference was observed in patients receiving docetaxel- or vinorelbine-based therapy (Figs. 1C and 1D).

In multivariate analysis adjusted for gender, smoking history, and stage of disease, RRM1 expression emerged as an independent predictive factor for PFS in patients receiving gemcitabine-based therapy (95% CI: 1.135–2.907, *P = *0.013).

### Tumor Response and PFS According to Chemotherapy Regimen

**Figure 2:**
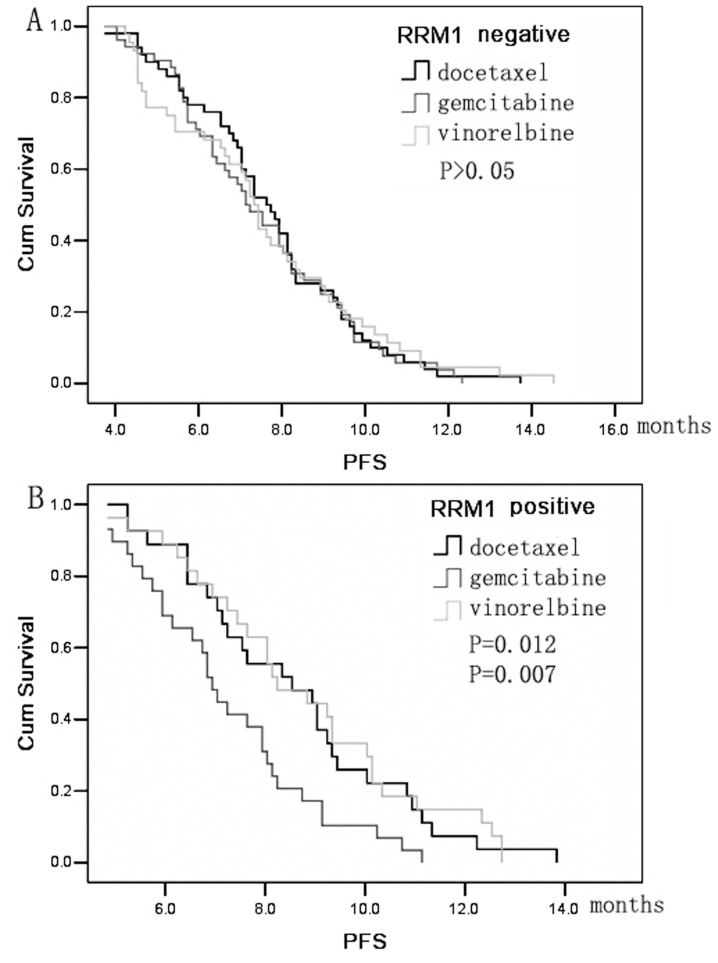
Kaplan-Meier curve of progression-free survival (PFS) according to chemotherapy regimen. (A) PFS for patients with RRM1-negative tumors. (B) PFS for patients with RRM1-positive tumors.

In patients with RRM1-negative tumors, no differences were observed in terms of RR, DCR, or PFS among patients that received gemcitabine-, docetaxel-, or vinorelbine-based therapies. However, in patients with RRM1-positive tumors, the DCR of patients receiving docetaxel or vinorelbine was higher than that of patients receiving gemcitabine (81.5% and 81.5% vs. 55.2%, respectively; *P = *0.047 and *P = *0.047) (Table 3). In addition, docetaxel and vinorelbine showed a longer PFS than gemcitabine-based chemotherapy (8.9 months and 9.1 months vs. 7.6 months, respectively; *P = *0.012 and *P = *0.007) (Figs. 2A and 2B).

## Discussion

In the present study, we analyzed 229 patients with NSCLC who had received carboplatin-based doublet chemotherapy. In patients receiving gemcitabine-based therapy, the DCR and PFS in patients with RRM1-negative tumors was significantly higher than in RRM1-positive cases, and multivariate analysis showed that RRM1 expression was an independent predictive factor for outcome. RRM1 overexpression in tumor tissue may induce resistance to gemcitabine-based therapy. Ribonucleotide reductase (RR) is an essential enzyme for DNA synthesis, and is inhibited by the active metabolite of gemcitabine, difluorideosycytidine 5-diphosphate. RRM1 depletes difluorideosycytidine 5-diphosphate and promotes DNA synthesis, thereby enabling tumor survival. In studies with lung cancer cell lines, RRM1 overexpression is associated with resistance to gemcitabine therapy [13], [18]. Consistently, clinical studies have also suggested that overexpression of RRM1 correlates with resistance to gemcitabine-based therapy [19], [20]. Conversely, low RRM1 mRNA expression was associated with a high response rate [21]. These studies demonstrate that RRM1 could be a predictive marker of the response to gemcitabine-based chemotherapy in patients with NSCLC [22].

The present study also showed that the DCR was higher in RRM1-positive patients that received docetaxel or vinorelbine, rather than gemcitabine-based therapy. In addition, docetaxel and vinorelbine each showed a longer PFS than gemcitabine-based therapy. Simon et al. used RRM1 and ERCC1 as molecular determinants, and found that RRM1- and ERCC1-tailored selection of first-line therapy could improve response, overall survival (OS), and PFS over standard treatments in patients with NSCLC [23]. These studies suggest that responses to cytotoxic chemotherapy vary greatly in patients with NSCLC, and individualized therapy based on RRM1 expression may help improve the efficacy of chemotherapeutic agents [24]. Our research was performed retrospectively, and this is the major limitation of the study. However, the current results provide new information and further insight that can assist clinicians in selecting appropriate and individualized chemotherapy for patients with NSCLC based on RRM1 expression.

Several molecular markers have been used as predictive markers of the response to chemotherapy in NSCLC patients. ERCC1 has been used for the prediction of platinum sensitivity in the treatment of NSCLC [6]–[8]. Park et al. analyzed 217 patients with NSCLC who had received gemcitabine- or taxane-based chemotherapy, and found that taxane was associated with a higher response than gemcitabine treatment in patients with EGFR mutations [9]. Another study found that low thymidylate synthase (TS) expression is significantly associated with better clinical outcomes in non-squamous NSCLC patients who were treated with pemetrexed-based chemotherapy [25]. Therefore, more prospectively designed studies with combined detection of these markers (RRM1, ERCC1, EGFR, and TS) will provide valuable information that will ultimately be used to determine preferable therapeutic approaches for individual patients with NSCLC.

In conclusion, the results of this study suggest that negative RRM1 expression in advanced NSCLC is associated with a higher response rate to gemcitabine-based chemotherapy. Moreover, RRM1 may be used as a predictive marker for conventional chemotherapy regimens involving gemcitabine, docetaxel, and vinorelbine. Additional prospective studies are needed to evaluate the effect of RRM1 expression on the response to various chemotherapeutic regimens in patients with NSCLC.
